# Impact of Anatomical and Viability-Guided Completeness of Revascularization on Clinical Outcomes in Ischemic Cardiomyopathy

**DOI:** 10.1016/j.jacc.2024.04.043

**Published:** 2024-07-23

**Authors:** Saad M. Ezad, Margaret McEntegart, Matthew Dodd, Matthaios Didagelos, Novalia Sidik, Matthew Li Kam Wa, Holly P. Morgan, Antonis Pavlidis, Roshan Weerackody, Simon J. Walsh, James C. Spratt, Julian Strange, Peter Ludman, Amedeo Chiribiri, Tim Clayton, Mark C. Petrie, Peter O’Kane, Divaka Perera, REVIVED-BCIS2 Investigators, Divaka Perera, Amedeo Chiribiri, Gerry Carr-White, Antonis Pavlidis, Simon Redwood, Brian Clapp, Aldo Rinaldi, Haseeb Rahman, Natalia Briceno, Sophie Arnold, Amy Raynsford, Mark Petrie, Margaret McEntegart, Stuart Watkins, Aadil Shaukat, Paul Rocchiccioli, Louise Cowan, Roshan Weerackody, Ceri Davies, Elliot Smith, Bhavik Modi, Peter O’Kane, Jehangir Din, Jonathon Hinton, John Greenwood, Jonathan Blaxill, Abdul Mozid, Michelle Anderson, Lana Dixon, Simon Walsh, Mark Spence, Patricia Glover, Richard Edwards, Adam McDiarmid, Mohaned Egred, Hannah Stevenson, George Amin-Youssef, Ajay Shah, Theresa McDonagh, Jonathan Byrne, Nilesh Pareek, Jonathan Breeze, Anthony Gershlick, Gerald McCann, Andrew Ladwiniec, Iain Squire, Donna Alexander, Kalpa De Silva, Julian Strange, Tom Johnson, Angus Nightingale, Laura Gallego, James Spratt, Claudia Cosgrove, Rupert Willia, Sam Firoozi, Pitt Lim, Dwayne Conway, Peter Swoboda, Paul Brooksby, James Cotton, Richard Horton, Stella Metherell, Kai Hogrefe, Adrian Cheng, Sian Sidgwick, Tim Lockie, Niket Patel, Roby Rakhit, Fozia Ahmed, Cara Hendry, Farzin Fath-Odoubadi, Douglas Fraser, Mamas Mamas, Miles Behan, Alan Japp, Nicholas Jenkins, Sam McClure, Karen Martin, Eltigani Abdelaal, Jaydeep Sarma, Sanjay Sastry, Jo Riley, Pradeep Magapu, Rod Stables, David Wright, Michael Mahmoudi, Andrew Flett, Nick Curzen, Sam Gough, Zoe Nicholas, Andrew Ludman, Hibba Kurdi, Sam Keenan, Kevin Thorpe, Prithwish Banerjee, Luke Tapp, Abeesh Panicker, Mark de Belder, Jeet Thambyrajah, Neil Swanson, Neville Kukreja, Mary Lynch, Girish Viswanathan, Elaine Jones, Sarah Norman, Helen Routledge, Jasper Trevelyan, Nick Pegge, Sukhbir Dhamrait, Tim Wells, Manas Sinha, Gavin Galasko, Christopher Cassidy, Tim Edwards, Javed Iqbal, Fraser Witherow, Kaeng Lee, James Beattie, Mike Pitt, Julian Gunn, Abdallah Al-Mohammad, Helen Denney, Huw Griffiths, Paul Kalra, Tim Gray, Jolanta Sobolewska, Steve Ramcharitar, Laura McCafferty, Thomas Martin, John Irving, Zaid Iskandar, Jason Glover, James Beynon, Maurice Pye, Simon Megarry, Paul Das, Chris Bellamy

**Affiliations:** a British Heart Foundation Centre of Research Excellence at the School of Cardiovascular and Metabolic Medicine & Sciences, King’s College London, London, United Kingdom; b West of Scotland Heart and Lung Centre, Golden Jubilee National Hospital, Clydebank, United Kingdom; c Columbia University Medical Center, New York, New York, USA; d Clinical Trials Unit, London School of Hygiene & Tropical Medicine, London, United Kingdom; e Guy’s and St Thomas’ NHS Foundation Trust, London, United Kingdom; f Barts Health NHS Trust, London, United Kingdom; g Belfast Health and Social Care NHS Trust, Belfast, United Kingdom; h St George’s Hospital, University of London, London, United Kingdom; i University Hospitals Bristol NHS Foundation Trust, Bristol, United Kingdom; j Institute of Cardiovascular Sciences, University of Birmingham, Birmingham, United Kingdom; k Department of Biomedical Engineering and Imaging Sciences, King’s College London, London, United Kingdom; l School of Cardiovascular and Medical Sciences, British Heart Foundation Glasgow Cardiovascular Research Centre, University of Glasgow, Glasgow, United Kingdom; m University Hospitals Dorset NHS Foundation Trust, Bournemouth, United Kingdom

**Keywords:** complete revascularization, heart failure, left ventricular dysfunction, percutaneous coronary intervention, stable coronary artery disease

## Abstract

**Background:**

Complete revascularization of coronary artery disease has been linked to improved outcomes in patients with preserved left ventricular (LV) function.

**Objectives:**

This study sought to identify the impact of complete revascularization in patients with severe LV dysfunction.

**Methods:**

Patients enrolled in the REVIVED-BCIS2 (Revascularization for Ischemic Ventricular Dysfunction) trial were eligible if baseline/procedural angiograms and viability studies were available for analysis by independent core laboratories. Anatomical and viability-guided completeness of revascularization were measured by the coronary and myocardial revascularization indices (RI_coro_ and RI_myo_), respectively, where RI_coro_ = (change in British Cardiovascular Intervention Society Jeopardy score [BCIS-JS]) / (baseline BCIS-JS) and RI_myo_= (number of revascularized viable segments) / (number of viable segments supplied by diseased vessels). The percutaneous coronary intervention (PCI) group was classified as having complete or incomplete revascularization by median RI_coro_ and RI_myo_. The primary outcome was death or hospitalization for heart failure.

**Results:**

Of 700 randomized patients, 670 were included. The baseline BCIS-JS and SYNTAX (Synergy Between PCI With Taxus and Cardiac Surgery) scores were 8 (Q1-Q3: 6-10) and 22 (Q1-Q3: 15-29), respectively. In those patients assigned to PCI, median RI_coro_ and RI_myo_ values were 67% and 85%, respectively. Compared with the group assigned to optimal medical therapy alone, there was no difference in the likelihood of the primary outcome in those patients receiving complete anatomical or viability-guided revascularization (HR: 0.90; 95% CI: 0.62-1.32; and HR: 0.95; 95% CI: 0.66-1.35, respectively). A sensitivity analysis by residual SYNTAX score showed no association with outcome.

**Conclusions:**

In patients with severe LV dysfunction, neither complete anatomical nor viability-guided revascularization was associated with improved event-free survival compared with incomplete revascularization or treatment with medical therapy alone. (Revascularization for Ischemic Ventricular Dysfunction) [REVIVED-BCIS2]; NCT01920048)

Treating as many diseased major coronary arteries as possible is a cornerstone of contemporary revascularization, and the perceived ability to achieve this goal often affects the choice of revascularization method, namely, percutaneous coronary intervention (PCI) or coronary artery bypass grafting.^1^ Incomplete revascularization has been associated with an increased incidence of death, myocardial infarction, and need for repeat revascularization.^2^ However, almost the entire evidence base for targeting complete revascularization has been derived from patients with good left ventricular function. Furthermore, although the treatment of critical coronary artery disease can be directly translated to myocardial benefit in patients with preserved left ventricular function, a more nuanced approach needs to be used when evaluating completeness of revascularization in ischemic cardiomyopathy, an approach that integrates the viability of subtended myocardial territories as well as the severity of coronary artery disease.

The premise that PCI is beneficial in ischemic cardiomyopathy is based on 2 key underlying principles. First, hibernation is an adaptive or maladaptive state in response to repeated episodes of ischemia, which is designed to preserve myocyte integrity at the expense of contractile function, thus resulting in viable but dysfunctional myocardium. Second, revascularization may reverse hibernation by relieving the supply-demand mismatch, thereby leading to angina relief, recovery in left ventricular function, and improved clinical outcomes.^3^ Whether the premise of complete revascularization holds true in this context remains unknown. This prespecified analysis of REVIVED-BCIS2 (Revascularization for Ischemic Ventricular Dysfunction; NCT01920048) therefore sought to explore the relationship between the extent of core laboratory–adjudicated anatomical and viability-guided revascularization and outcomes in ischemic cardiomyopathy.

## Methods

The design and primary results of the REVIVED-BCIS2 have been previously published.^4^^,^^5^ Briefly, eligible participants with ischemic left ventricular dysfunction (ejection fraction ≤35%), extensive coronary artery disease denoted by a British Cardiovascular Interventional Society jeopardy score (BCIS-JS) ≥6, and demonstrable viability in ≥4 myocardial segments amenable to revascularization were randomized 1:1 to a strategy of either PCI combined with optimal medical therapy (OMT) (the PCI group) or OMT alone (the OMT group) at 40 centers in the United Kingdom (Supplemental Appendix). Although complete anatomical revascularization was not mandated in REVIVED-BCIS2, the protocol recommended revascularization of all major proximal coronary vessels and side branches ≥2.5 mm subtending viable myocardium. This included vessels with chronic total occlusion (CTO), when specialist CTO operators anticipated a high likelihood of reopening these vessels successfully.^5^ Clinical outcomes were adjudicated by a blinded clinical events committee, and left ventricular ejection fraction was independently reported by a core laboratory with readers blinded to treatment assignment, outcome data, and temporal sequence of the echocardiograms. The trial protocol was approved by the UK Health Research Authority, and all participants provided written informed consent.

Pre-PCI BCIS-JS and SYNTAX (Synergy Between PCI With Taxus and Cardiac Surgery) scores were ascertained from all participants who had an angiogram available for analysis by an independent coronary angiography core laboratory (Golden Jubilee National Hospital, Glasgow, United Kingdom). For participants assigned to the PCI group, post-PCI BCIS-JS and residual SYNTAX score (rSS) were also calculated following the final planned PCI procedure as reported by investigators. The core laboratory reported lesion severity by visual assessment, with significance defined at ≥70% luminal stenosis for non–left main stem stenoses and ≥50% for left main stem stenoses for calculation of the BCIS-JS.^6^^,^^7^ Successful revascularization of a vessel was defined as a <30% diameter residual stenosis with normal (TIMI [Thrombolysis In Myocardial Infarction] flow grade 3) flow at the end of PCI. Anatomical completeness of revascularization was described by the coronary revascularization index (RI_coro_) calculated as: ([Pre-PCI BCIS-JS] − [Post-PCI BCIS-JS]) / (Pre-PCI BCIS-JS) × 100. A sensitivity analysis was preformed using rSS to define anatomical completeness of revascularization, with rSS dichotomized as ≤8 or >8.^8^ The RI_coro_ was 0 for all participants in the OMT group.

In patients who underwent viability assessment by cardiac magnetic resonance (CMR) or dobutamine stress echocardiography (DSE), images were independently analyzed by dedicated core laboratories (CMR core laboratory at King’s College London, London, United Kingdom, and DSE core laboratory at King’s Health Partners, United Kingdom) blinded to treatment assignment and outcome data. Myocardial viability was described using the American Heart Association (AHA) 17 segment model.^9^ For the current analysis, a segment was classified as viable if wall motion was normal at rest, or if dysfunctional at rest, when there was <50% transmural late gadolinium scar on CMR or the presence of contractile reserve on DSE. Segments that did not meet these criteria were classified as nonviable.

AHA myocardial segments were co-registered to a coronary artery on the basis of the highest percentage chance of that segment being subtended by the relevant coronary artery^10^ (Supplemental Figure 1). The status of each AHA myocardial segment was then classified as being supplied by an artery with significant disease and revascularized (REVASC), supplied by an artery with significant disease but not revascularized (NO REVASC), or not supplied by an artery with significant disease (NO DISEASE). The myocardial revascularization index (RI_myo_) was calculated as: (REVASC / [REVASC + NO REVASC]) × 100, limited to the number of viable myocardial segments (Supplemental Figure 2). Participants assigned to OMT were assumed to have an RI_myo_ of 0. Participants in the PCI group who did not have pre-PCI and post-PCI angiography and a CMR or DSE viability test of sufficient quality for core laboratory analysis were excluded from this analysis.

The primary outcome was a composite of death from any cause or hospitalization for heart failure over all follow-up (minimum follow-up was 24 months). Secondary outcomes were all-cause death, cardiovascular death, hospitalization for heart failure, and improvement in left ventricular function at 6 months (defined as a greater than the median absolute change in left ventricular ejection fraction on echocardiography).

### Statistical analysis

The statistical analysis plan was finalized before the lock and unblinding of angiographic core laboratory data. A formal power calculation was not performed for this secondary analysis. A Cox proportional hazards model was constructed to assess the relationship between each of RI_coro_, RI_myo_, and the primary outcome, adjusted for age, sex, previous heart failure hospitalization, the presence of diabetes, chronic renal failure, left ventricular ejection fraction, extent of coronary disease, and the presence of at least 1 CTO; for RI_coro_, the model was also adjusted for the extent of nonviable myocardium. The proportionality assumption of Cox models was assessed by visual examination and, for the primary analyses, using Schoenfeld residuals. Results are reported as estimates with corresponding 95% CIs, the widths of which have not been adjusted for multiplicity. Participants in the OMT group without baseline angiography available for core laboratory analysis were included in the Cox models for RI_coro_ and RI_myo_ because the revascularization index in these cases was assumed to be 0. Missing values of left ventricular ejection fraction and the adjustment variables (Supplemental Table 1) were imputed using a multiple imputation model with chained equations that included randomized treatment, age, sex, history of heart failure hospitalization, diabetes, estimated glomerular filtration rate, death during follow-up, hospitalization for heart failure during follow-up, and baseline, 6-month, and 12-month left ventricular ejection fractions. Twenty imputations were performed and effect estimates combined using Rubin’s rules.

RI_coro_ and RI_myo_ were considered continuous variables, and the median values of each were also used to dichotomously define complete vs incomplete anatomical and viability-guided revascularization, respectively; Kaplan-Meier curves were created for each of the latter comparisons.

Logistic regression models were created and adjusted for the same baseline covariates previously discussed, to explore the relationships among RI_coro_, RI_myo_, and improvement in left ventricular function. These analyses were restricted to participants who were alive at 6 months, with missing ejection fraction values imputed as previously described. Results are presented as mean ± SD or median (Q1-Q3). All analyses were conducted using Stata software version 17.0 (StataCorp).

## Results

**Figure 1:**
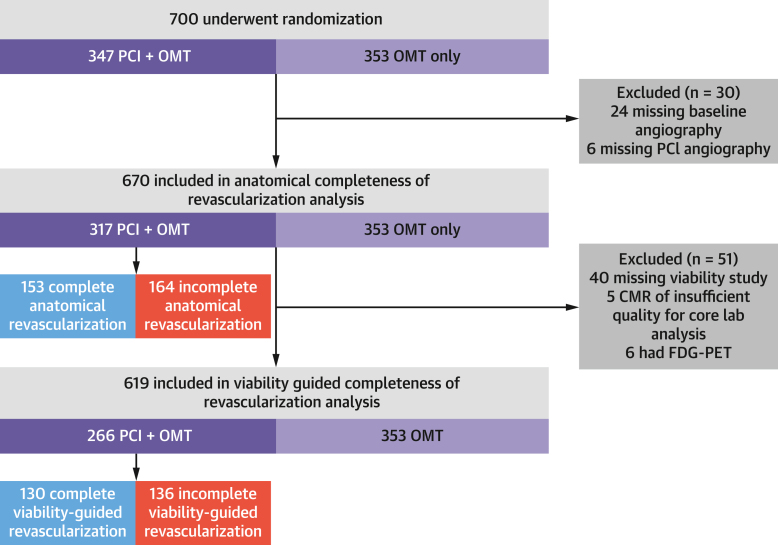
Study Consolidated Standards of Reporting Trials (CONSORT) Diagram A total of 18 patients in the optimal medical therapy (OMT) arm had missing baseline angiography but were included in completeness of revascularization analyses because the revascularization index was assumed to be 0. CMR = cardiac magnetic resonance; FDG-PET = fluorodeoxyglucose positron emission tomography; PCI = percutaneous coronary intervention.

Of the 700 participants in REVIVED-BCIS2, 670 were included were included in the anatomical completeness of revascularization analysis (317 assigned to PCI and 353 assigned to OMT), and 619 were included in the viability-guided completeness of revascularization analysis (266 in the PCI group and 353 in the OMT group) (Figure 1). Baseline clinical, demographic, anatomical, and viability characteristics were well matched between the groups (Table 1). Prescription rates of guideline-directed medical therapy were similar at baseline and at follow-up (Supplemental Table 2).

**Table 1:** Baseline Characteristics of Participants in Anatomical and Viability-Guided Completeness of Revascularization Analyses

	REVIVED-BCIS2 Trial (n = 700)	Anatomical CoR Analysis (n = 670)	Viability-Guided CoR Analysis (n = 619)
Age, y	69.4 ± 9.1	69.2 ± 9.1	69.1 ± 9.0
Male	614 (87.7)	587 (87.6)	544 (87.9)
Body mass index, kg/m^2^	28.0 (24.7-31.7)	28.1 (24.9-31.9)	28.1 (24.9-32.0)
Diabetes	289 (41.3)	277 (41.3)	260 (42.0)
Hypertension	391 (55.9)	378 (56.5)	348 (56.3)
Current or previous smoker	510 (72.9)	490 (73.1)	454 (73.3)
Cerebrovascular disease	84 (12.0)	81 (12.1)	70 (11.3)
Peripheral vascular disease	94 (13.4)	90 (13.4)	85 (13.7)
Race^a^			
Asian	49 (7.0)	47 (7.0)	40 (6.5)
Black	6 (0.9)	6 (0.9)	6 (1.0)
Mixed, other, or not reported	11 (1.6)	11 (1.6)	10 (1.6)
White	634 (90.6)	606 (90.4)	563 (91.0)
History of myocardial infarction	372 (53.1)	356 (53.1)	327 (52.8)
Hospitalization for heart failure in previous 2 y	233 (33.3)	221 (33.0)	213 (34.4)
Previous PCI	142 (20.3)	136 (20.3)	121 (19.5)
Previous CABG	34 (4.9)	33 (4.9)	31 (5.0)
CCS angina class			
0	464 (66.6)	448 (67.2)	418 (67.9)
1	143 (20.5)	137 (20.5)	126 (20.5)
2	75 (10.8)	70 (10.5)	61 (9.9)
3	14 (2.0)	12 (1.8)	11 (1.8)
4	1 (0.1)	0 (0.0)	0 (0.0)
NYHA functional class			
I	126 (18.1)	121 (18.2)	115 (18.7)
II	387 (55.7)	373 (56.1)	347 (56.5)
III	172 (24.7)	163 (24.5)	145 (23.6)
IV	10 (1.4)	8 (1.2)	7 (1.1)
Cardiac medication			
RAAS inhibitor	584 (83.5)	557 (83.3)	511 (82.7)
Beta-blocker	634 (90.6)	608 (90.7)	561 (90.6)
MRA	364 (49.4)	332 (49.6)	308 (49.8)
Baseline BCIS jeopardy score^b^	8 (6-10)	8 (6-10)	8 (6-10)
Post-PCI BCIS jeopardy score	2 (0-4)	2 (0-4)	2 (0-4)
Baseline SYNTAX score	22.0 (15.0-28.5)	22.0 (15.0-28.5)	22.0 (15.0-29.0)
Residual SYNTAX score	8.0 (2.0-14.0)	8.0 (2.0-14.0)	8.0 (2.0-14.0)
ICD with or without CRT at randomization	148 (21.1)	140 (20.9)	129 (20.8)
Left main coronary artery disease	95 (13.6)	88 (13.2)	85 (13.8)
LVEF, %^c^	31.9 ± 9.9	31.9 ± 9.8	32.1 ± 9.8
Viability test			
CMR	479 (78.5)	458 (78.2)	453 (78.0)
DSE	131 (21.5)	128 (21.8)	128 (22.0)
Number of viable segments	7 (4-10)	7 (4-10)	7 (4-10)

Values are mean ± SD, n (%), or median (Q1-Q3).

BCIS = British Cardiovascular Intervention Society; CABG = coronary artery bypass grafting; CCS = Canadian Cardiovascular Society; CMR = cardiovascular magnetic resonance; CoR = completeness of revascularization; CRT = cardiac resynchronization therapy; CTO = chronic total occlusion; DSE = dobutamine stress echocardiography; ICD = implantable cardioverter-defibrillator; LVEF = left ventricular ejection fraction; MRA = mineralocorticoid receptor antagonist; PCI = percutaneous coronary intervention; Q = quartile; RAAS = renin-angiotensin-aldosterone system; REVIVED-BCIS2 = Revascularization for Ischemic Ventricular Dysfunction; SYNTAX = Synergy Between PCI With Taxus and Cardiac Surgery.

a Race as self-reported by participants using options defined by the investigators.

b The BCIS jeopardy score is a quantification of the extent of myocardial jeopardy relating to clinically significant coronary artery stenoses. The score ranges from 0 (no significant coronary disease) to 12 (disease jeopardizing the whole left ventricular myocardium). The score presented is as calculated by the angiography core laboratory.

c Baseline left ventricular ejection fraction measured by the blinded echocardiography core laboratory.

### Anatomical completeness of revascularization

A total of 658 participants had baseline coronary angiography available for core laboratory analysis. The median baseline BCIS-JS and SYNTAX scores were 8 (Q1-Q3: 6-10) and 22 (Q1-Q3: 15-29), respectively. A total of 351 (53%) patients had at least 1 CTO, and 340 (52%) had at least 1 lesion with moderate to severe angiographic calcification. Of the 317 patients assigned to PCI (and included in this analysis), 62 (20%) had at least 1 CTO successfully treated. In the PCI group, the median post-PCI BCIS-JS was 2 (Q1-Q3: 0-4) representing a median reduction of 6 (Q1-Q3: 2-8) (Supplemental Table 3), thus resulting in an RI_coro_ of 67% (Q1-Q3: 50%-100%) (Supplemental Table 4). Core laboratory–reported RI_coro_ showed good agreement with site-reported RI_coro_, with only 6.7% of measurements lying outside the limits of agreement (Supplemental Figure 3). Patients achieving complete anatomical revascularization tended to be younger, were less likely to have a history of myocardial infarction, and had lower baseline BCIS-JS and SYNTAX scores as compared with patients who received incomplete revascularization (Supplemental Table 5).

**Figure 2:**
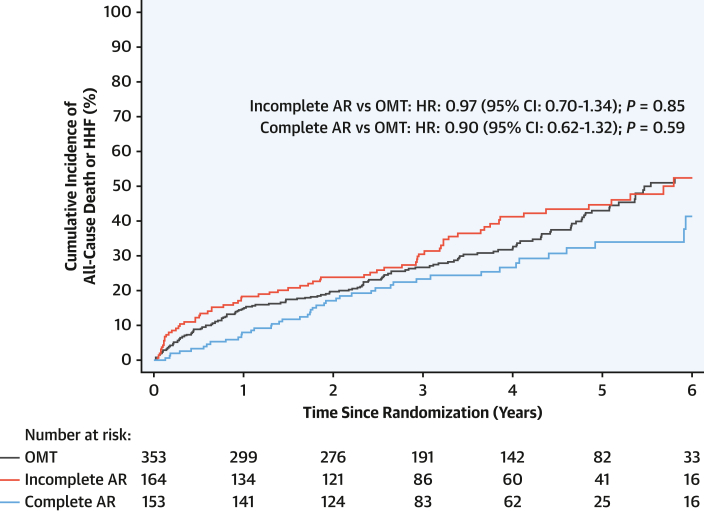
Anatomical Completeness of Revascularization vs OMT Kaplan-Meier plot of the primary outcome (death or hospitalization for heart failure [HHF]). The presented HRs for comparisons are adjusted. Incomplete anatomical revascularization (AR) vs optimal medical therapy (OMT): unadjusted HR: 1.13; 95% CI: 0.85-1.51; *P* = 0.40. Complete anatomical revascularization vs optimal medical therapy: unadjusted HR: 0.75; 95% CI: 0.54-1.06; *P* = 0.10.

Compared with OMT alone, complete anatomical revascularization did not reduce the primary outcome (adjusted HR: 0.90; 95% CI: 0.62-1.32; *P* = 0.59) (Figure 2). A sensitivity analysis categorizing patients by rSS also found no difference in primary outcome between those patients who had an rSS ≤8 compared with patients assigned to OMT alone (HR: 1.00; 95% CI: 0.69-1.44; *P* > 0.99) (Supplemental Table 6, Supplemental Figure 4). Similarly, there was no association between achieving complete anatomical revascularization and improvement in left ventricular function (OR: 0.94; 95% CI: 0.54-1.64; *P* = 0.82) or occurrence of any of the other secondary outcomes (Central Illustration**,** Supplemental Table 7). When treating RI_coro_ as a continuous variable in the PCI group only, there appeared to be a reduction in the incidence of the primary outcome with increasing degrees of revascularization (HR: 0.92 per 10% increase in RI_coro_; 95% CI: 0.87-0.97; *P* = 0.003), but this association was no longer apparent after adjustment for baseline risk (HR: 0.94 per 10% increase in RI_coro_; 95% CI: 0.88-1.01 per 10% increase in RI_coro_; *P* = 0.10) (Supplemental Table 8).

**Central Illustration:**
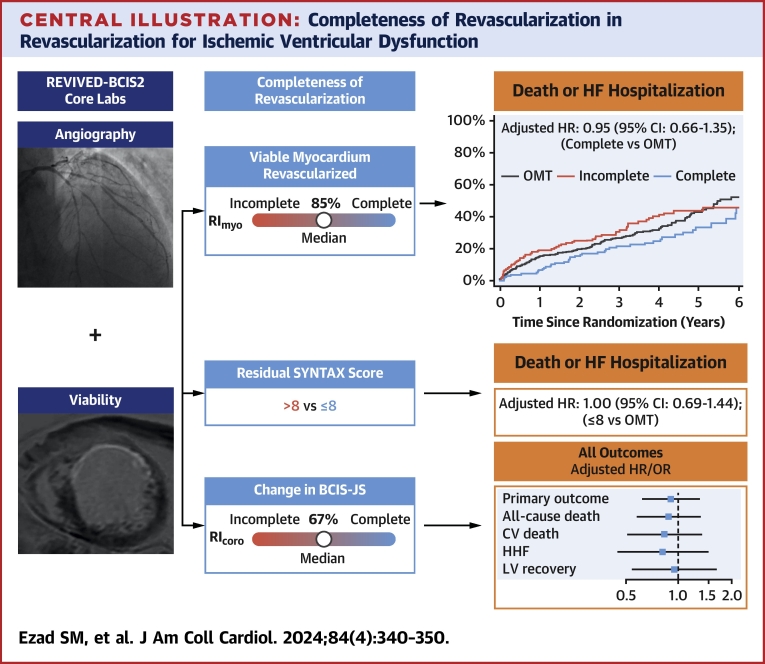
Completeness of Revascularization in Revascularization for Ischemic Ventricular Dysfunction Core laboratory–analyzed coronary angiography and cardiac magnetic resonance were used to define anatomical and viability-guided completeness of revascularization. Primary and secondary outcomes are presented for those patients achieving complete revascularization vs optimal medical therapy (OMT). BCIS-JS = British Cardiovascular Intervention Society Jeopardy score; CV = cardiovascular; HF = heart failure; HHF = hospitalization for heart failure; LV = left ventricular; REVIVED-BCIS2 = Revascularization for Ischemic Ventricular Dysfunction; RI_coro_ = coronary revascularization index; RI_myo_ = myocardial revascularization index; SYNTAX = Synergy Between PCI [Percutaneous Coronary Intervention] With Taxus and Cardiac Surgery.

### Viability-guided completeness of revascularization

**Figure 3:**
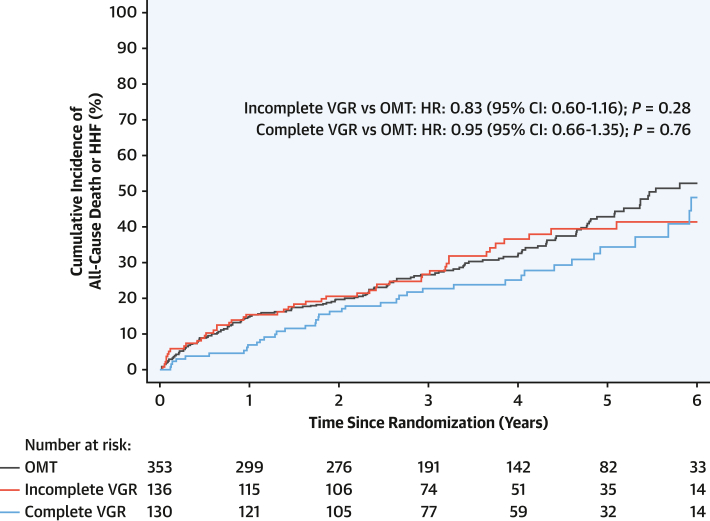
Viability-Guided Completeness of Revascularization vs OMT Kaplan-Meier plot of the primary outcome (death or hospitalization for heart failure [HHF]). The presented HRs for comparisons are adjusted. Incomplete viability-guided revascularization (VGR) vs optimal medical therapy (OMT): unadjusted HR: 0.93; 95% CI: 0.67-1.30; *P* = 0.68. Complete viability-guided revascularization vs optimal medical therapy: unadjusted HR: 0.80; 95% CI: 0.56-1.13; *P* = 0.20.

**Figure 4:**
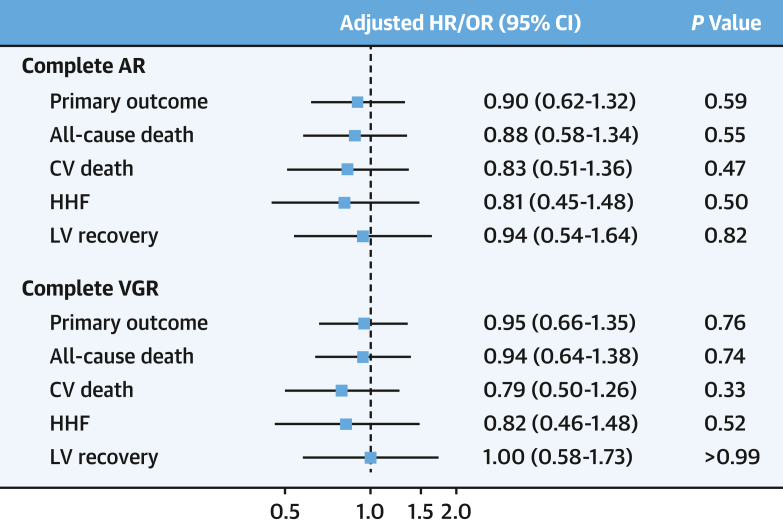
Primary and Secondary Outcomes for Complete Revascularization Forest plot presenting the treatment effect of complete anatomical revascularization (AR) and viability-guided revascularization (VGR) on primary and prespecified secondary outcomes. CV = cardiovascular; HHF = hospitalization for heart failure; LV = left ventricular.

In the cohort included in this analysis, the median number of segments that were viable and subtended by significant coronary artery disease was 5 (Q1-Q3: 3-7). In the PCI group, 3 (Q1-Q3: 1-6) segments were revascularized per participant, thus yielding a median RI_myo_ of 85% (Q1-Q3: 60%-100%) (Supplemental Table 4)**.** Complete viability-guided revascularization by PCI was not associated with a reduction in the occurrence of the primary outcome (HR: 0.95; 95% CI: 0.66-1.35; *P* = 0.76) (Figure 3) or any of the secondary outcomes (Figure 4, Supplemental Table 9). No difference was found in the rate of left ventricular improvement in those patients who achieved complete viability-guided revascularization (OR: 1.00; 95% CI: 0.58-1.73; *P* > 0.99). Similar to anatomically incomplete revascularization, those patients who underwent incomplete viability-guided revascularization were older and had more extensive and complex baseline disease, including a higher incidence of left main stem disease (Supplemental Table 10).

A sensitivity analysis using a late gadolinium transmurality cutoff of 25% to define viability similarly found no interaction with the primary outcome in the patients who achieved complete viability-guided revascularization (HR: 1.02; 95% CI: 0.72-1.44; *P* = 0.93) or with any of the secondary outcomes (Supplemental Table 11). When viability was considered as a continuous variable, there was no evidence for an association with the primary outcome per 10% increase in RI_myo_ (unadjusted HR: 0.98; 95% CI: 0.91-1.04; *P* = 0.47; adjusted HR: 1.00; 95% CI: 0.93-1.08; *P* = 0.97) (Supplemental Table 8).

### Quality of life and completeness of revascularization

The baseline summary Kansas City Cardiomyopathy Questionnaire (KCCQ) score was lowest among those patients achieving incomplete revascularization (Supplemental Table 12). As compared with OMT alone, achieving complete anatomical revascularization was associated with a nonsignificant improvement (adjusted mean difference: 4.6; 95% CI: −0.2 to 9.5; *P* = 0.06) in KCCQ score at 2 years (Supplemental Table 12). A similar trend toward improvement was observed with those patients achieving complete viability-guided revascularization (adjusted mean difference: 3.9; 95% CI: −0.9 to 8.6; *P* = 0.11).

## Discussion

In this prespecified analysis of REVIVED-BCIS2 using core laboratory analyses of baseline and postprocedural angiograms as well as viability studies, we did not find an association between the extent of anatomical or viability-guided completeness of revascularization and the treatment effect of PCI with respect to the occurrence of death or hospitalization for heart failure or the likelihood of left ventricular recovery. Core laboratory–adjudicated RI_coro_ was comparable to previously published site-reported RI_coro_^4^ and was lower than RI_myo_, findings reflecting the large burden of nonviable myocardium, which is a key determinant of which diseased vessels are chosen as targets for revascularization. This also explains why increasing degrees of anatomical revascularization initially appeared to be associated with increased benefit, but this association was no longer evident when the extent of nonviable myocardium was taken into consideration.

The strongest evidence in support of complete revascularization comes from randomized studies of patients with multivessel disease presenting with acute coronary syndromes.^11^, ^12^, ^13^ In the COMPLETE (Complete vs Culprit-only Revascularization to Treat Multi-vessel Disease After Early PCI for STEMI [ST-Segment Elevation Myocardial Infarction]) trial, the benefit was primarily driven by a reduction in subsequent myocardial infarction as opposed to cardiovascular death, a finding suggesting that the risk relates to the likelihood of atherosclerotic plaque rupture, which can, in turn, be modulated by revascularization.^13^ Conversely, in the CULPRIT-SHOCK (Culprit Lesion Only PCI Versus Multivessel PCI in Cardiogenic Shock) trial, which enrolled patients with acute left ventricular dysfunction following myocardial infarction, multivessel PCI was associated with worse outcomes as compared with culprit lesion–only PCI, a finding that may reflect the need to balance acute procedural risks against potential long-term benefits.^14^ No prospective randomized studies of complete vs incomplete revascularization have been conducted to date in stable coronary artery disease. A post hoc secondary analysis of the ISCHEMIA (International Study of Comparative Health Effectiveness With Medical and Invasive Approaches) trial reported an apparent reduction in the rate of cardiovascular death and myocardial infarction in those patients with complete anatomical revascularization; however, these differences were no longer significant after adjustment for baseline characteristics.^15^ In this substudy, completeness of revascularization was not randomized but was at the discretion of the attending clinicians; patients who underwent incomplete revascularization were found to be more comorbid, with more extensive and complex coronary artery disease. Similarly, a post hoc analysis of the patients assigned to the PCI arm of the SYNTAX trial found that patients with an rSS >8 (representing incomplete anatomical revascularization) were associated with an increased risk of all-cause death (35.3% vs 8.5% at 5 years; *P* < 0.001) with a more pronounced effect in the subgroup with impaired left ventricular function,^16^ although patients who had an rSS >8 were older and had higher rates of diabetes, peripheral vascular disease, and CTOs, thus resulting in higher baseline SYNTAX scores and higher EuroSCORE (European System for Cardiac Operative Risk Evaluation) values.

Given that the patient’s baseline risk strongly influences (and is usually inversely related to) the degree of revascularization achieved, such nonrandomized comparisons of complete vs incomplete revascularization are heavily confounded and are not fully accounted for by techniques such as propensity matching or modeling. We also found that patients undergoing incomplete revascularization had lower baseline KCCQ scores, more comorbidities, and more extensive and complex coronary disease. The finding of similar event rates in this cohort, despite their higher baseline risk, provides further indirect evidence that incomplete (anatomical or viability-guided) revascularization does not confer a prognostic penalty in patients with severe ischemic left ventricular dysfunction.

The distinction between anatomical and functional completeness of revascularization also merits further consideration. In stable coronary syndromes, in patients with preserved left ventricular function, these metrics may be discordant because it is well recognized that there is an imperfect correlation between the anatomical severity of a coronary artery lesion (most commonly visualized by angiography) and its ability to cause ischemia.^17^ There is a growing body of evidence that better clinical outcomes can be achieved with a functional (ischemia-guided) revascularization strategy than with a strategy that is based on anatomical (angiographically apparent) coronary artery disease, even though the former usually results in revascularization of fewer vessels and lesions.^18^ When treating patients with stable ischemic cardiomyopathy, the viability of subtended myocardium is a unique consideration. Only critically diseased vessels that subtend viable myocardium are usually considered for revascularization because this is believed to be the primary substrate for regional ischemic ventricular dysfunction, whereas there is no evidence that revascularization of scarred and nonviable regions is of benefit.

To capture these specific goals, we have used a novel measure of viability-guided revascularization, the RI_myo_, which expresses completeness of revascularization in relation to the extent of viable myocardium that is supplied by diseased coronary arteries. By this measure, the degree of viability-guided revascularization achieved in the PCI arm of REVIVED-BCIS2 was high (approximately 85%), but we found no evidence that complete viability-guided revascularization provided benefit beyond that of incomplete revascularization or OMT alone. These findings suggest that, in established ischemic cardiomyopathy, the risk of subsequent adverse events arises from the state of the myocardium rather than from plaque rupture and also that reversal of advanced hibernation cannot be achieved by revascularization alone. These data corroborate the REVIVED-BCIS2 viability analysis, which demonstrated that the key determinant of clinical outcomes and ventricular recovery was the extent of nonviable myocardium.^19^

### Study limitations

First, we did not randomize to a strategy of complete revascularization vs incomplete revascularization, and hence our results are prone to selection bias, which has affected other observational studies in this arena. However, the finding of similar event rates in those patients who had complete vs incomplete revascularization, despite a more adverse risk profile in the latter, adds further weight to our conclusion that completeness of revascularization does not affect outcomes in these patients. Second, co-registration of AHA segments to a coronary vessel territory was standardized on the basis of coronary dominance. An approach customized to individual coronary anatomy may have allowed improved accuracy of co-registration, but it would be prone to subjectivity and hence be less reproducible. Third, for simplicity of analysis and presentation, we used a binary classification of complete vs incomplete even though a spectrum of revascularization exists. However, our findings were congruent even when RI_coro_ and RI_myo_ were analyzed as continuous variables. Fourth, we did not systematically capture intracoronary physiology and imaging data, and hence the core laboratory analysis is purely based on visual assessment of angiograms, whereas these data will have been used by clinicians to inform the BCIS-JS calculation and to guide management of patients assigned to PCI, as recommended by the trial protocol. Finally, we assessed only revascularization with PCI. Coronary artery bypass grafting represents a fundamentally different method of achieving revascularization that could be associated with different outcomes.

## Conclusions

This study does not show a difference in event-free survival or frequency of improved left ventricular function in patients with stable coronary disease and severe impairment of left ventricular function who were assigned to PCI and subsequently underwent complete revascularization compared with patients who were assigned to PCI but underwent incomplete revascularization or patients who were assigned to OMT alone. This finding is consistent whether completeness of revascularization was classified by the overall angiographic burden of coronary disease or the extent of revascularization of viable myocardium.

Perspectives **COMPETENCY IN MEDICAL KNOWLEDGE:** In patients with severe ischemic left ventricular dysfunction, complete revascularization by PCI, compared with incomplete revascularization, did not reduce the incidence of death or heart failure hospitalization.

**TRANSLATIONAL OUTLOOK:** Randomized trials are needed to clarify the impact of complete revascularization compared with incomplete revascularization by PCI in patients with stable coronary artery disease.

## Funding Support and Author Disclosures

The trial was funded by the National Institute for Health and Care Research (UK) Health Technology Assessment Program (NIHR 10/57/67); and the present work was supported by the British Heart Foundation (FS/CRTF/21/24118, RE/18/2/34213 and RE/18/6/34217). The authors have reported that they have no relationships relevant to the contents of this paper to disclose.
