# Supplementary material for: Impact of Anatomical and Viability-Guided Completeness of Revascularization on Clinical Outcomes in Ischemic Cardiomyopathy
Source: J Am Coll Cardiol. 2024 Jul 23;84(4):340–50. doi: 10.1016/j.jacc.2024.04.043 (PMC11250908; doi:10.1016/j.jacc.2024.04.043)
Supplement: Supplementary Figures 1-4 and Supplementary Tables 1-12 [file mmc1.docx]

**Supplementary Material**

Contents

[1. REVIVED Sites and Investigators 3](#_Toc167374768)

[2. Trial Organization and Oversight 5](#_Toc167374769)

[Core Laboratories 5](#_Toc167374770)

[Cardiac MRI Core Laboratory 5](#_Toc167374771)

[Dobutamine Stress Echocardiography Core Laboratory 5](#_Toc167374772)

[Echocardiography Core Laboratory 5](#_Toc167374773)

[Angiographic Core Laboratory 5](#_Toc167374774)

[Committees and Oversight 6](#_Toc167374775)

[Trial Steering Committee 6](#_Toc167374776)

[Data and Safety Monitoring Committee 6](#_Toc167374777)

[Clinical Events Committee 6](#_Toc167374778)

[Trial Statisticians 7](#_Toc167374779)

[3. Figures 8](#_Toc167374780)

[Figure S1 – Co-registration of coronary lesions to American Heart Association myocardial segments 8](#_Toc167374781)

[Figure S2 – Example of coronary and myocardial revascularization index calculation 9](#_Toc167374782)

[Figure S3 – Comparison of core lab vs site reported anatomical completeness of revascularization (PCI Group) 10](#_Toc167374783)

[Figure S4 – Impact of anatomical completeness of revascularization by residual SYNTAX score on the primary outcome 11](#_Toc167374784)

[4. Tables 12](#_Toc167374785)

[Table S1 – Imputed missing data 12](#_Toc167374786)

[Table S2 – Utilization of guideline directed medical therapy 13](#_Toc167374787)

[Table S3 – Core-lab adjudicated BCIS-JS 14](#_Toc167374788)

[Table S4 – Core-lab adjudicated anatomical and viability-guided completeness of revascularization 14](#_Toc167374789)

[Table S5 – Comparison of baseline characteristics in those achieving complete vs incomplete anatomical revascularization 15](#_Toc167374790)

[Table S6 – Primary and clinical secondary outcomes by residual SYNTAX score 17](#_Toc167374791)

[Table S7 – Primary and clinical secondary outcomes by anatomical completeness of revascularization 17](#_Toc167374792)

[Table S8 – Relationship between completeness of revascularization and outcomes (with RI_coro_ and RI_myo_ as continuous variables) 18](#_Toc167374793)

[Table S9 – Primary and secondary outcomes by viability-guided completeness of revascularization (50% late gadolinium enhancement threshold) 18](#_Toc167374794)

[Table S10 - Comparison of baseline characteristics in those achieving complete vs incomplete viability-guided revascularization 19](#_Toc167374795)

[Table S11 – Primary and secondary outcomes by viability guided completeness of revascularization (25% late gadolinium enhancement threshold) 21](#_Toc167374796)

[Table S12 – Change in 2-year summary KCCQ score by anatomical and viability-guided completeness of revascularization 22](#_Toc167374797)

# REVIVED Sites and Investigators

| **Center** | **Principal Investigator** | **Site team** |
| --- | --- | --- |
| Guy’s & St Thomas’ Hospital | Prof Divaka Perera | Prof Amedeo Chiribiri  Prof Gerry Carr-White  Dr Antonis Pavlidis  Prof Simon Redwood  Dr Brian Clapp  Prof Aldo Rinaldi  Dr Haseeb Rahman  Dr Natalia Briceno  Ms Sophie Arnold  Ms Amy Raynsford |
| Golden Jubilee National Hospital, Glasgow | Prof Mark Petrie | Dr Margaret McEntegart  Dr Stuart Watkins  Dr Aadil Shaukat  Dr Paul Rocchiccioli  Ms Louise Cowan |
| Barts Heart Centre, London | Dr Roshan Weerackody | Dr Ceri Davies  Dr Elliot Smith  Dr Bhavik Modi |
| Royal Bournemouth Hospital | Dr Peter O’Kane | Dr Jehangir Din  Dr Jonathon Hinton |
| Leeds General Infirmary | Prof John Greenwood | Dr Jonathan Blaxill  Dr Abdul Mozid  Ms Michelle Anderson |
| Royal Victoria Hospital, Belfast | Dr Lana Dixon | Dr Simon Walsh  Dr Mark Spence  Ms Patricia Glover |
| Freeman Hospital, Newcastle | Dr Richard Edwards | Dr Adam McDiarmid  Dr Mohaned Egred  Ms Hannah Stevenson |
| King’s College Hospital, London | Dr George Amin-Youssef | Prof Ajay Shah  Prof Theresa McDonagh  Dr Jonathan Byrne  Dr Nilesh Pareek  Mr Jonathan Breeze |
| Glenfield Hospital, Leicester | Prof Anthony Gershlick | Prof Gerald McCann  Dr Andrew Ladwiniec  Prof Iain Squire  Ms Donna Alexander |
| Bristol Royal Infirmary | Dr Kalpa De Silva | Dr Julian Strange  Dr Tom Johnson  Dr Angus Nightingale  Ms Laura Gallego |
| St George’s Hospital, London | Prof James Spratt | Dr Claudia Cosgrove  Dr Rupert Williams  Dr Sam Firoozi  Dr Pitt Lim |
| Pinderfields Hospital, Wakefield | Dr Dwayne Conway | Dr Peter Swoboda  Dr Paul Brooksby |
| New Cross Hospital, Wolverhampton | Dr James Cotton | Dr Richard Horton  Ms Stella Metherell |
| Kettering General Hospital | Dr Kai Hogrefe | Dr Adrian Cheng  Ms Sian Sidgwick |
| Royal Free Hospital, London | Dr Tim Lockie | Dr Niket Patel  Dr Roby Rakhit |
| Manchester Royal Infirmary | Dr Fozia Ahmed | Dr Cara Hendry  Dr Farzin Fath-Odoubadi  Dr Douglas Fraser  Dr Mamas Mamas |
| Royal Infirmary of Edinburgh | Dr Miles Behan | Dr Alan Japp |
| Sunderland Royal Hospital | Dr Nicholas Jenkins | Dr Sam McClure  Ms Karen Martin |
| Wythenshawe Hospital | Dr Eltigani Abdelaal | Dr Jaydeep Sarma  Dr Sanjay Sastry  Dr Jo Riley |
| Liverpool Heart and Chest Hospital | Dr Pradeep Magapu | Prof Rod Stables  Dr David Wright |
| Southampton General Hospital | Dr Michael Mahmoudi | Dr Andrew Flett  Prof Nick Curzen  Ms Sam Gough  Ms Zoe Nicholas |
| Royal Devon & Exeter Hospital | Dr Andrew Ludman | Dr Hibba Kurdi  Ms Sam Keenan  Mr Kevin Thorpe |
| University Hospitals Coventry & Warwickshire | Prof Prithwish Banerjee | Dr Luke Tapp  Mr Abeesh Panicker |
| The James Cook University Hospital, Middlesbrough | Dr Mark de Belder | Dr Jeet Thambyrajah  Dr Neil Swanson |
| Lister Hospital, Stevenage | Dr Neville Kukreja | Dr Mary Lynch |
| Derriford Hospital, Plymouth | Dr Girish Viswanathan | Ms Elaine Jones  Ms Sarah Norman |
| Worcestershire Acute Hospitals | Dr Helen Routledge | Dr Jasper Trevelyan |
| Worthing Hospital | Dr Nick Pegge | Dr Sukhbir Dhamrait |
| Salisbury District Hospital | Dr Tim Wells | Dr Manas Sinha |
| Blackpool Victoria Hospital | Dr Gavin Galasko | Dr Christopher Cassidy |
| Dorset County Hospital | Dr Tim Edwards | Dr Javed Iqbal  Dr Fraser Witherow |
| Birmingham Heartlands Hospital | Dr Kaeng Lee | Dr James Beattie  Dr Mike Pitt |
| Northern General Hospital, Sheffield | Dr Julian Gunn | Dr Abdallah Al-Mohammad  Ms Helen Denney |
| Queen Alexandra Hospital, Portsmouth | Dr Huw Griffiths | Prof Paul Kalra |
| Royal Oldham Hospital | Dr Tim Gray | Dr Jolanta Sobolewska |
| Great Western Hospital, Swindon | Dr Steve Ramcharitar | Ms Laura McCafferty |
| Ninewells Hospital, Dundee | Dr Thomas Martin | Dr John Irving  Dr Zaid Iskandar |
| Basingstoke & North Hampshire Hospital | Dr Jason Glover | Dr James Beynon |
| The York Hospital | Mr Maurice Pye | Dr Simon Megarry |
| North Wales Cardiac Centre | Dr Paul Das | Dr Chris Bellamy |
|  |  |  |

# Trial Organization and Oversight

## Core Laboratories

### Cardiac MRI Core Laboratory

Prof Amedeo Chiribiri (Lead; Reader), King’s College London

Dr Pier Giorgio Masci (Reader), King’s College London

Dr Sohaib Nazir (Reader), King’s College London

Dr Jennifer Silva, King’s College London

Dr Ebraham Alskaf, King’s College London

Dr Holly Morgan, King’s College London

### Dobutamine Stress Echocardiography Core Laboratory

Prof Roxy Senior (Lead; Reader), Royal Brompton Hospital, London

Dr Alexandros Papachristidis (Reader), King’s College Hospital, London

Dr Navtej Chahal (Reader), Royal Brompton Hospital, London

Dr Rajdeep Khattar (Reader), Royal Brompton Hospital, London

Dr Saad Ezad, King’s College London

### Echocardiography Core Laboratory

Dr Stam Kapetenakis (Lead), Guy’s and St Thomas’ Hospital, London

Ms Jane Draper (Reader), Guy’s and St Thomas’ Hospital, London

Ms Sheila Subbiah (Reader), Guy’s and St Thomas’ Hospital, London

Ms Annabel Oraa (Reader), Guy’s and St Thomas’ Hospital, London

Ms Olga Khaleva (Reader), Guy’s and St Thomas’ Hospital, London

Dr Haotian Gu (Reader), Guy’s and St Thomas’ Hospital, London

Dr Sarah Blake (Reader), Guy’s and St Thomas’ Hospital, London

Ms Emily Denman (Reader), King’s College Hospital, London

Ms Almira Whittaker (Reader), King’s College Hospital, London

Ms Marilou Huang (Reader), King’s College Hospital, London

Ms Sandya Nandakumar (Reader), King’s College Hospital, London

Dr Joseph Okafor (Reader), Guy’s and St Thomas’ NHS Foundation Trust, London

Dr Oleksandr Danylenko (Reader), Guy’s and St Thomas’ NHS Foundation Trust, London

### Angiographic Core Laboratory

Dr Margaret McEntegart (Lead), Golden Jubilee National Hospital, Glasgow

Dr Matthaios Didangelos (Reader), Golden Jubilee National Hospital, Glasgow

Dr Novalia Sidik (Reader), Golden Jubilee National Hospital, Glasgow

## Committees and Oversight

### Trial Steering Committee

Prof Andrew Clark, Chair of Clinical Cardiology, Castle Hill Hospital, Hull (Chair)

Mrs Helen Williams, Pharmacist, NHS Southwark Clinical Commissioning Group, London

Dr Pablo Perel, Epidemiologist, London School of Hygiene & Tropical Medicine

Dr David Walker, Consultant Cardiologist, Conquest Hospital, St. Leonards-on-Sea

Prof Rod Stables, Consultant Cardiologist, Liverpool Heart and Chest Hospital

Prof Divaka Perera, Chief Investigator, King’s College London

Ms Liz Bestic, Patient, Carer and Public representative

Mrs Paula Young, Patient, Carer and Public representative

Mrs Helen Datta, Patient, Carer and Public representative

Mr Jeremy Dearling, Patient, Carer and Public representative

### Data and Safety Monitoring Committee

Prof Peter Ludman, Consultant Cardiologist, Queen Elizabeth Hospital, Birmingham (Chair)

Dr Suzanna Hardman, Consultant Cardiologist, The Whittington Hospital, London

Dr Louise Brown, Senior Statistician, MRC Clinical Trials Unit at University College London

### Clinical Events Committee

Prof Roxy Senior, Professor of Cardiology, Royal Brompton Hospital, London (Chair)

Dr Zaheer Yousef, Consultant Cardiologist, University Hospital of Wales

Dr Rajan Sharma, Consultant Cardiologist, St George’s Hospital, London

Dr Shazia Hussain, Consultant Cardiologist, University Hospitals of Leicester NHS Trust

Dr Stephen Hoole, Consultant Cardiologist, Royal Papworth Hospital

Dr Ninian Lang, Reader in Cardiology, University of Glasgow

Dr Kieran Docherty, Clinical Lecturer in Cardiology, University of Glasgow

Dr Roy Gardner, Consultant Cardiologist, Golden Jubilee National Hospital, Glasgow

Prof Andrew Sharp, Consultant Cardiologist, University Hospital of Wales

Dr Ricardo Petraco, Consultant Cardiologist, Imperial College Healthcare NHS Trust

Dr Vasileios Panoulas, Consultant Cardiologist, Royal Brompton and Harefield Hospitals

Dr Andreas Schuster, Consultant Cardiologist, Universitätsmedizin Göttingen, Germany

Dr Kaleab Asrress, Consultant Cardiologist, Bankstown-Lidcombe Hospital, Australia

Dr Matthew Lee, Clinical Lecturer in Cardiology, University of Glasgow

Prof Pardeep Jhund, Professor of Cardiology and Epidemiology, University of Glasgow

Dr Eugene Connolly, Director, Global Clinical Trial Partners, Glasgow

Prof Raj Kharbanda, Consultant Cardiologist, John Radcliffe Hospital, Oxford

Ms Farandeep Dhaliwal, London School of Hygiene & Tropical Medicine (Admin)

### Trial Statisticians

Ms Joanne Dobson, London School of Hygiene & Tropical Medicine

Mr Matthew Dodd, London School of Hygiene & Tropical Medicine

Prof Tim Clayton, London School of Hygiene & Tropical Medicine

# Figures

## Figure S1 – Co-registration of coronary lesions to American Heart Association myocardial segments

Figure S1A – Right dominant circulation


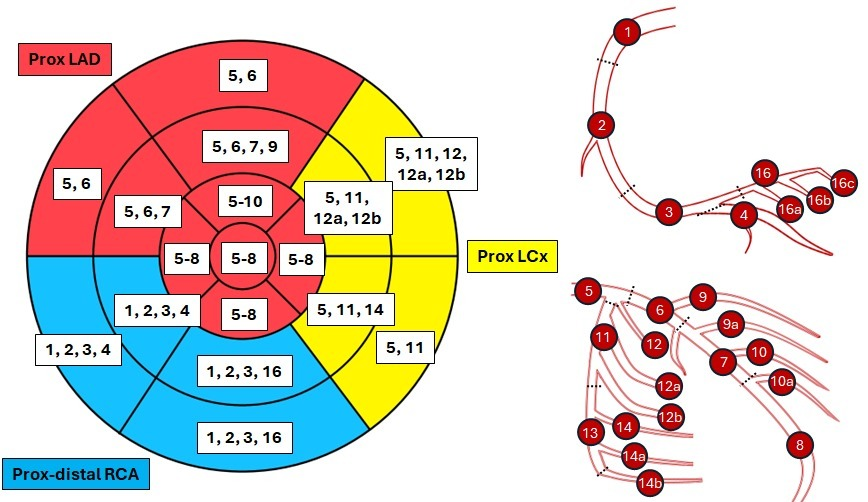


Figure S1B – Left dominant circulation


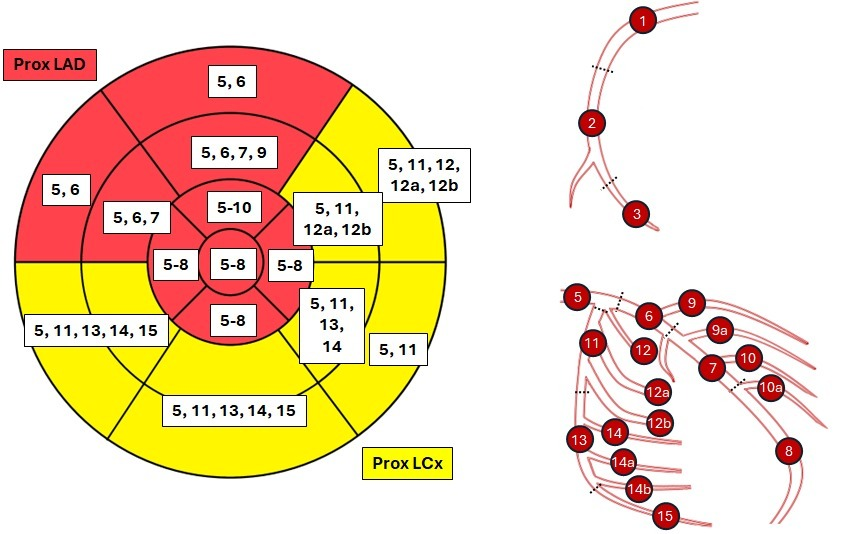


Each coronary lesion with a visual diameter stenosis of >70% was assigned to a SYNTAX segment by core lab readers. SYNTAX segments were then linked to American Heart Association segments as demonstrated in these polar maps depending on coronary dominance.

## Figure S2 – Example of coronary and myocardial revascularization index calculation

**
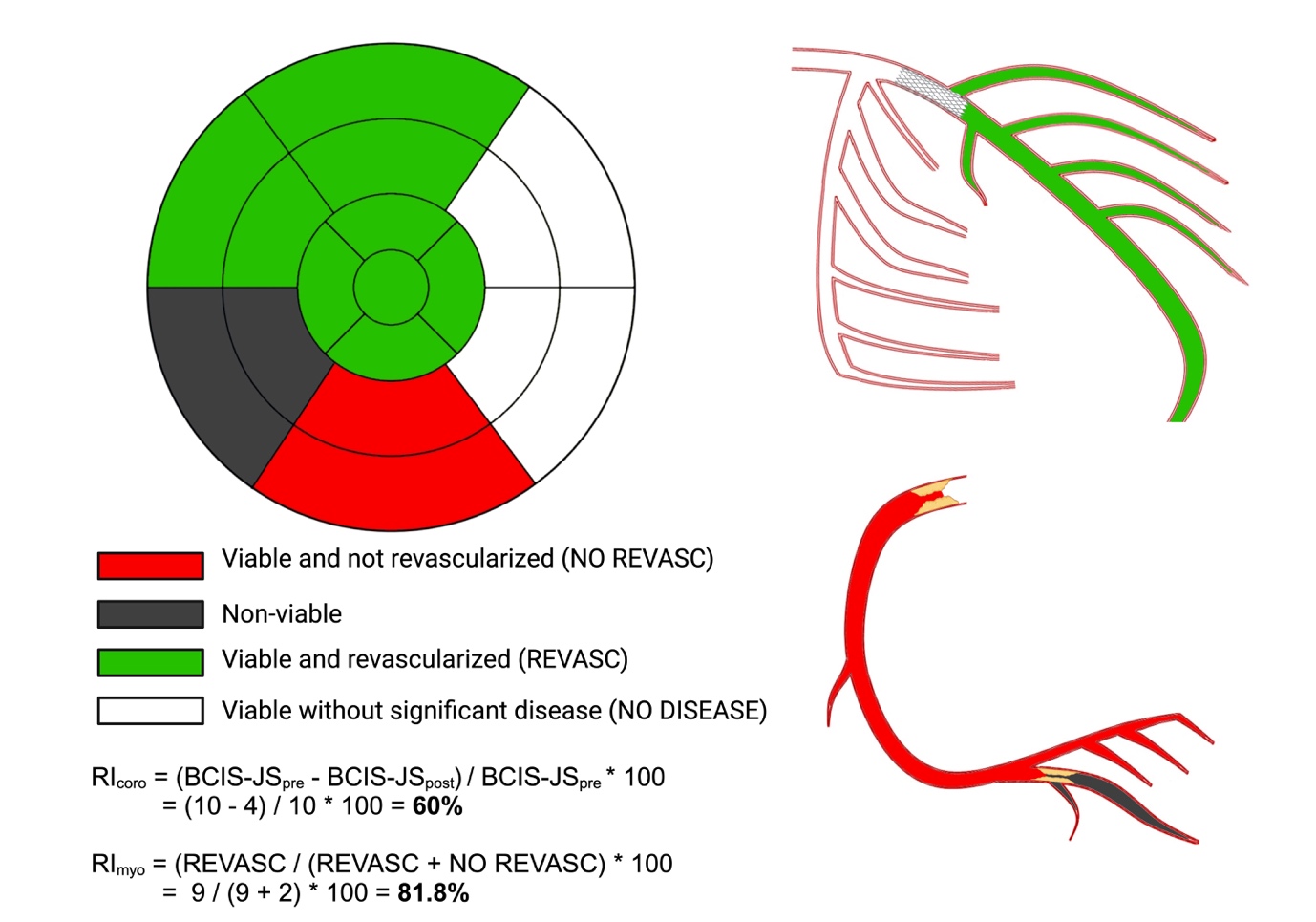
**

BCIS-JS - British Cardiovascular Interventional Society jeopardy score, RI_coro_ – Coronary revascularization index, RI_myo_ – Myocardial revascularization index

## Figure S3 – Comparison of core lab vs site reported anatomical completeness of revascularization (PCI Group)

Bland-Altman plot comparing core lab and site reported RI_coro_. Mean difference observed was -5.3%.

RI_coro_ – Coronary revascularization index

## Figure S4 – Impact of anatomical completeness of revascularization by residual SYNTAX score on the primary outcome

The Kaplan-Meir plot presents the adjusted HR for comparisons. rSS ≤ 8 vs OMT: Unadjusted HR = 0.79 (95% CI 0.57 to 1.08), p=0.14. rSS >8 vs OMT: Unadjusted HR = 1.13 (95% CI 0.84 to 1.53), p=0.42

CI – confidence interval, HR – hazard ratio, OMT – optimal medical therapy, rSS – residual SYNTAX score

# Tables

## Table S1 – Imputed missing data

| **Anatomical completeness of revascularization analysis (n=670)** |  |
| --- | --- |
| **Variable** | Number of missing values |
| eGFR | 9 |
| LVEF at baseline | 153 |
| LVEF at 6 monyhd | 154 |
| LVEF at 12 months | 164 |
|  |  |
| **Viability guided completeness of revascularization analysis (n=619)** |  |
| **Variable** | Number of missing values |
| eGFR | 9 |
| LVEF at baseline | 137 |
| LVEF at 6 months | 146 |
| LVEF 12 months | 150 |

eGFR – estimated glomerular filtration rate, LVEF – left ventricular ejection fraction

## Table S2 – Utilization of guideline directed medical therapy

|  | **REVIVED trial**  **(n=700)** | **Anatomical CoR analysis**  **(n=670)** | **Viability-guided CoR analysis (n=619)** |
| --- | --- | --- | --- |
| **6 months** |  |  |  |
| RAAS inhibitor | 493/647 (76.2) | 475/625 (76.0) | 438/579 (75.7) |
| Beta blocker | 604/647 (93.4) | 584/625 (93.4) | 542/579 (93.6) |
| Mineralocorticoid receptor antagonist | 343/647 (53.1) | 333/624 (53.4) | 312/578 (54.0) |
|  |  |  |  |
| **1 year** |  |  |  |
| RAAS inhibitor | 466/625 (74.6) | 451/606 (74.4) | 414/561 (73.8) |
| Beta blocker | 585/625 (93.6) | 566/606 (93.4) | 527/561 (93.9) |
| Mineralocorticoid receptor antagonist | 340/624 (54.5) | 332/605 (54.9) | 310/560 (55.4) |
|  |  |  |  |
| **2 years** |  |  |  |
| RAAS inhibitor | 369/567 (65.1) | 356/550 (64.7) | 326/509 (64.1) |
| Beta blocker | 529/569 (93.0) | 512/552 (92.8) | 477/511 (93.4) |
| Mineralocorticoid receptor antagonist | 315/567 (55.6) | 310/550 (56.4) | 288/510 (56.5) |

CoR completeness of revascularization, RAAS renin angiotensin aldosterone system.

## Table S3 – Core-lab adjudicated BCIS-JS

|  | **OMT arm** | **PCI arm only** | |
| --- | --- | --- | --- |
| **Baseline BCIS-JS**^†^ | **Baseline** | **Baseline** | **Post-PCI** |
| 0 | 2/335 (0.6) | 4/317 (1.3) | 122/317 (38.5) |
| 2 | 10/335 (3.0) | 13/317 (4.1) | 56/317 (17.7) |
| 4 | 27/335 (8.1) | 26/317 (8.2) | 68/317 (21.5) |
| 6 | 84/335 (25.1) | 72/317 (22.7) | 49/317 (15.5) |
| 8 | 67/335 (20.0) | 68/317 (21.5) | 12/317 (3.8) |
| 10 | 68/335 (20.3) | 56/317 (17.7) | 5/317 (1.6) |
| 12 | 77/335 (23.0) | 78/317 (24.6) | 5/317 (1.6) |
| Median (IQR) | 8 (6 to 10) | 8 (6 to 10) | 2 (0 to 4) |

† The British Cardiovascular Intervention Society jeopardy score (BCIS-JS) is a quantification of the extent of myocardial jeopardy relating to clinically significant coronary artery stenoses. The score ranges from 0 (no significant coronary disease) to 12 (disease jeopardizing the whole left ventricular myocardium).

## Table S4 – Core-lab adjudicated anatomical and viability-guided completeness of revascularization

| **Revascularization index** | **RI_coro_ n (%)** | **RI_myo_ n (%)** |
| --- | --- | --- |
| ≤20% | 28/317 (8.8) | 22/266 (8.3) |
| 21 to 40% | 47/317 (14.8) | 18/266 (6.8) |
| 41 to 60% | 62/317 (19.6) | 29/266 (10.9) |
| 61 to 80% | 50/317 (15.8) | 54/266 (20.3) |
| 81 to 99% | 12/317 (3.8) | 32/266 (12.0) |
| 100% | 118/317 (37.2) | 111/266 (41.7) |
| Median (IQR) | 66.7 (50.0 to 100.0) | 84.6 (60.0 to 100.0) |
|  |  |  |

RI_coro_ – Coronary revascularization index. RI_myo_ – Myocardial revascularization index.

## Table S5 – Comparison of baseline characteristics in those achieving complete vs incomplete anatomical revascularization

|  | **Optimal medical therapy**  **(N=353)** | **Incomplete anatomical revascularization (RI_coro_ ≤66.7)**  **(N=164)** | **Complete anatomical revascularization (RI_coro_ >66.7)**  **(N=153)** | P-value^d^ |
| --- | --- | --- | --- | --- |
| Age, mean (SD), years | 68.8 (9.1) | 70.8 ± 8.4 | 68.7 ± 9.6 | 0.03 |
| Male sex (%) | 312 (88.4) | 143 (87.2) | 132 (86.3) | 0.81 |
| Body-mass index (IQR) | 27.9 (24.9 to 32.0) | 27.7 (24.4 to 31.6) | 28.7 (25.0 to 32.0) | 0.50 |
| Diabetes (%) | 153 (43.3) | 61 (37.2) | 63 (41.2) | 0.47 |
| Hypertension (%) | 207 (58.8) | 91 (55.5) | 80 (52.3) | 0.57 |
| Current or previous smoker (%) | 267 (75.6) | 117 (71.3) | 106 (69.3) | 0.69 |
| Cerebrovascular disease (%) | 46 (13.0) | 21 (12.8) | 14 (9.2) | 0.29 |
| Peripheral vascular disease (%) | 46 (13.0) | 26 (15.9) | 18 (11.8) | 0.29 |
| Race (%)^a^  Asian  Black  Mixed, other or not reported  White | 17 (4.8)  3 (0.8)  5 (1.4)  328 (92.9) | 19 (11.6)  2 (1.2)  1 (0.6)  142 (86.6) | 11 (7.2)  1 (0.7)  5 (3.3)  136 (88.9) | 0.17 |
| History of myocardial infarction (%) | 197 (55.8) | 94 (57.3) | 65 (42.5) | 0.01 |
| Hospitalization for heart failure in prior 2 years (%) | 121 (34.3) | 48 (29.3) | 52 (34.0) | 0.37 |
| Previous PCI (%) | 76 (21.5) | 28 (17.1) | 32 (20.9) | 0.38 |
| Previous CABG (%) | 22 (6.2) | 9 (5.5) | 2 (1.3) | 0.06 |
| CCS Angina Class  0  1  2  3  4 | 236 (67.2)  75 (21.4)  32 (9.1)  8 (2.3)  0 (0.0) | 102 (62.6)  35 (21.5)  23 (14.1)  3 (1.8)  0 (0.0) | 110 (71.9)  27 (17.6)  15 (9.8)  1 (0.7)  0 (0.0 | 0.32 |
| NYHA Class  I  II  III  IV | 57 (16.3)  191 (54.6)  96 (27.4)  6 (1.7) | 27 (16.6)  93 (57.1)  42 (25.8)  1 (0.6) | 37 (24.3)  89 (58.6)  25 (16.4)  1 (0.7) | 0.09 |
| ICD +/- CRT at randomization (%) | 71 (20.1) | 42 (25.6) | 27 (17.6) | 0.09 |
| Baseline BCIS jeopardy score, median (IQR)^b^ | 8 (6 to 10) | 10 (6 to 12) | 8 (6 to 10) | 0.0008 |
| Post-PCI BCIS jeopardy score, median (IQR) | - | 4 (4 to 6) | 0 (0 to 0) | <0.0001 |
| Baseline SYNTAX score, median (IQR) | 22 (15 to 29) | 23.5 (19.0 to 30.8) | 18.0 (13.0 to 24.5) | <0.0001 |
| Residual SYNTAX score, median (IQR) | - | 13.0 (8.3 to 20.0) | 1.0 (0.0 to 5.0) | <0.0001 |
| Left main coronary artery disease (%) | 45 (12.8) | 25 (15.2) | 18 (11.8) | 0.37 |
| Left ventricular ejection fraction, mean (SD), %^c^ | 31.9 ± 9.6 | 31.1 ± 9.1 | 32.8 ± 11.0 | 0.19 |
| Viability test (%)  CMR  DSE | 243 (77.1)  72 (22.9) | 116 (81.1)  27 (18.9) | 99 (77.3)  29 (22.7) | - |
| Number of viable segments (IQR) | 7 (4-10) | 6 (4-10) | 7 (5-11) | 0.21 |

^a^ Race as self-reported by participants using options defined by the investigators.

^b^ British Cardiovascular Intervention Society jeopardy score (BCIS-JS) as reported by angiography core laboratory.

^c^ Baseline left ventricular ejection fraction measured by the blinded echocardiography core laboratory

^d^ P-value denotes comparison between Incomplete vs complete anatomical revascularization groups

BCIS denotes British Cardiovascular Intervention Society, CABG coronary artery bypass grafting, CCS Canadian Cardiovascular Society, CMR cardiovascular magnetic resonance imaging, CRT cardiac resynchronization therapy, CTO chronic total occlusion, DSE dobutamine stress echocardiography, ICD implantable cardioverter defibrillator, IQR interquartile range, NYHA New York Heart Association, PCI percutaneous coronary intervention, RI_coro_ Coronary revascularization index.

## Table S6 – Primary and clinical secondary outcomes by residual SYNTAX score

|  | **Optimal medical therapy group^§^ (Reference)** | **rSS >8^§^** | **Hazard / Odds ratio***  **(95% CI)** | **p-value** | **rSS ≤8^§^** | **Hazard / Odds ratio***  **(95% CI)** | **p-value** |
| --- | --- | --- | --- | --- | --- | --- | --- |
| All-cause death or hospitalization for heart failure | 134 (38.0) | 61(42.7) | 0.88  (0.62 to 1.24) | 0.47 | 53 (30.8) | 1.00  (0.69 to 1.44) | >0.99 |
| All-cause death | 115 (32.6) | 52(36.4) | 0.81  (0.55 to 1.18) | 0.27 | 44 (25.6) | 0.99  (0.66 to 1.47) | 0.95 |
| Cardiovascular death | 88 (24.9) | 36 (25.2) | 0.68  (0.43 to 1.07) | 0.10 | 29 (16.9) | 0.90  (0.56 to 1.45) | 0.67 |
| Hospitalization for heart failure | 54 (15.3) | 23 (16.1) | 0.77  (0.44 to 1.37) | 0.37 | 24 (14.0) | 0.85  (0.48 to 1.52) | 0.58 |
| Improvement in left ventricular ejection fraction | 101 (50.2) | 44 (51.8) | 1.05  (0.57 to 1.94) | 0.87 | 48 (45.7) | 0.78  (0.45 to 1.35) | 0.37 |

CI – confidence interval; HR-hazard ratio; rSS – residual SYNTAX score

* Adjusted Hazard ratios calculated with OMT group as reference

**^§^** Event rate - n(%)

## Table S7 – Primary and clinical secondary outcomes by anatomical completeness of revascularization

|  | **Optimal medical therapy^§^ (Reference)** | **Complete anatomical revascularization^§^ (RI_coro_ >66.7)** | **Hazard / Odds ratio***  **(95% CI)** | **p-value** | **Incomplete anatomical revascularization^§^ (RI_coro_ ≤66.7)** | **Hazard / Odds ratio***  **(95% CI)** | **p-value** |
| --- | --- | --- | --- | --- | --- | --- | --- |
| All-cause death or hospitalization for heart failure | 134 (38.0) | 45 (29.4) | 0.90  (0.62 to 1.32) | 0.59 | 70 (42.7) | 0.97  (0.70 to 1.34) | 0.85 |
| All-cause death | 115 (32.6) | 36 (23.5) | 0.88  (0.58 to 1.34) | 0.55 | 60 (36.6) | 0.88  (0.62 to 1.25) | 0.48 |
| Cardiovascular death | 88 (24.9) | 24 (15.7) | 0.83  (0.51 to 1.36) | 0.47 | 41 (25.0) | 0.73  (0.48 to 1.12) | 0.15 |
| Hospitalization for heart failure | 54 (15.3) | 21 (13.7) | 0.81  (0.45 to 1.48) | 0.50 | 27 (16.5) | 0.84  (0.49 to 1.44) | 0.53 |
| Improvement in left ventricular ejection fraction | 101 (50.2) | 44 (46.8) | 0.94 (0.54 to 1.64) | 0.82 | 48 (49) | 0.85 (0.48 to 1.51) | 0.58 |

CI – confidence interval; HR-hazard ratio; RI_coro_ – Coronary revascularization index

* Adjusted Hazard/Odds ratios calculated with OMT group as reference

**^§^** Event rate - n(%)

## Table S8 – Relationship between completeness of revascularization and outcomes (with RI_coro_ and RI_myo_ as continuous variables)

| **Revascularization index** | **Outcome measure** | **Association**  Unadjusted HR/OR; 95% CI | **Association**  Adjusted HR/OR; 95% CI |
| --- | --- | --- | --- |
| **RI_coro_**  *per 10% increase (PCI arm only)* | Death or HHF | 0.92 (0.87 to 0.97) | 0.94 (0.88 to 1.01) |
|  | All-cause death | 0.92 (0.86 to 0.97) | 0.93 (0.86 to 1.01) |
|  | CV death | 0.91 (0.84 to 0.98) | 0.94 (0.86 to 1.04) |
|  | HHF | 0.96 (0.88 to 1.05) | 0.98 (0.87 to 1.10) |
|  | LV improvement | 1.05 (0.97 to 1.14) | 1.06 (0.95 to 1.18) |
|  |  |  |  |
| **RI_myo_**  *per 10% increase (PCI arm only)* | Death or HHF | 0.98 (0.91 to 1.04) | 1.00 (0.93 to 1.08) |
|  | All-cause death | 0.98 (0.91 to 1.06) | 1.00 (0.92 to 1.09) |
|  | CV death | 0.98 (0.90 to 1.07) | 1.01 (0.91 to 1.11) |
|  | HHF | 0.93 (0.84 to 1.03) | 0.97 (0.86 to 1.08) |
|  | LV improvement | 1.04 (0.95 to 1.14) | 1.03 (0.91 to 1.15) |

CI – confidence interval; CV- cardiovascular; HHF- hospitalization for heart failure; HR-hazard ratio; LV – left ventricle; OR - odds ratio; PCI – percutaneous coronary intervention; RI_coro_ – Coronary revascularization index; RI_myo_ – Myocardial revascularization index.

## Table S9 – Primary and secondary outcomes by viability-guided completeness of revascularization (50% late gadolinium enhancement threshold)

|  | **Optimal medical therapy^§^ (Reference)** | **Complete viability guided revascularization^§^ (RI_myo_ >84.6)** | **Hazard / Odds ratio***  **(95% CI)** | **p-value** | **Incomplete viability guided revascularization^§^ (RI_myo_ ≤84.6)** | **Hazard / Odds ratio***  **(95% CI)** | **p-value** |
| --- | --- | --- | --- | --- | --- | --- | --- |
| All-cause death or hospitalization for heart failure | 134 (38.0) | 42 (32.3) | 0.95  (0.66 to 1.35) | 0.76 | 49 (36.0) | 0.83  (0.60 to 1.16) | 0.28 |
| All-cause death | 115 (32.6) | 36 (27.7) | 0.94  (0.64 to 1.38) | 0.74 | 40 (29.4) | 0.77  (0.53 to 1.11) | 0.16 |
| Cardiovascular death | 88 (24.9) | 24 (18.5) | 0.79  (0.50 to 1.26) | 0.33 | 29 (21.3) | 0.71  (0.47 to 1.10) | 0.13 |
| Hospitalization for heart failure | 54 (15.3) | 15 (11.5) | 0.82  (0.46 to 1.48) | 0.52 | 18 (13.2) | 0.74  (0.43 to 1.27) | 0.27 |
| Improvement in left ventricular ejection fraction | 101 (50.2) | 40 (50.6) | 1.00  (0.58 to 1.73) | >0.99 | 44 (53.0) | 0.95  (0.54 to 1.67) | 0.86 |

CI – confidence interval; HR-hazard ratio; RI_myo_ – Myocardial revascularization index

* Adjusted Hazard ratios calculated with OMT group as reference

**^§^** Event rate - n(%)

## Table S10 - Comparison of baseline characteristics in those achieving complete vs incomplete viability-guided revascularization

|  | **Optimal medical therapy**  **(N=353)** | **Incomplete viability guided revascularization (RI_myo_ ≤86.7)**  **(N=136)** | **Complete viability guided revascularization (RI_myo_ >86.7)**  **(N=130)** | P-value^d^ |
| --- | --- | --- | --- | --- |
| Age, mean (SD), years | 68.8 (9.1) | 70.5 ± 8.3 | 68.3 ± 9.6 | 0.04 |
| Male sex (%) | 312 (88.4) | 122 (89.7) | 110 (84.6) | 0.21 |
| Body-mass index (IQR) | 27.9 (24.9 to 32.0) | 27.7 (24.2 to 30.9) | 28.7 (25.1 to 32.4) | 0.24 |
| Diabetes (%) | 153 (43.3) | 56 (41.2) | 51 (39.2) | 0.75 |
| Hypertension (%) | 207 (58.8) | 78 (57.4) | 63 (48.5) | 0.14 |
| Current or previous smoker (%) | 267 (75.6) | 95 (69.9) | 92 (70.8) | 0.87 |
| Cerebrovascular disease (%) | 46 (13.0) | 14 (10.3) | 10 (7.7) | 0.46 |
| Peripheral vascular disease (%) | 46 (13.0) | 23 (16.9) | 16 (12.3) | 0.29 |
| Race (%)^a^  Asian  Black  Mixed, other or not reported  White | 17 (4.8)  3 (0.8)  5 (1.4)  328 (92.9) | 14 (10.3)  1 (0.7)  3 (2.2)  118 (86.8) | 9 (6.9)  2 (1.5)  2 (1.5)  117 (90.0) | 0.71 |
| History of myocardial infarction (%) | 197 (55.8) | 79 (58.1) | 51 (39.2) | 0.002 |
| Hospitalization for heart failure in prior 2 years (%) | 121 (34.3) | 46 (33.8) | 46 (35.4) | 0.79 |
| Previous PCI (%) | 76 (21.5) | 24 (17.6) | 21 (16.2) | 0.75 |
| Previous CABG (%) | 22 (6.2) | 7 (5.1) | 2 (1.5) | 0.17 |
| CCS Angina Class  0  1  2  3  4 | 236 (67.2)  75 (21.4)  32 (9.1)  8 (2.3)  0 (0.0) | 92 (68.1)  30 (22.2)  11 (8.1)  2 (1.5)  0 (0.0) | 90 (69.2)  21 (16.2)  18 (13.8)  1 (0.8)  0 (0.0 | 0.31 |
| NYHA Class  I  II  III  IV | 57 (16.3)  191 (54.6)  96 (27.4)  6 (1.7) | 28 (20.69)  77 (57.5)  29 (21.6)  0 (0.0) | 30 (23.1)  79 (60.8)  20 (15.4)  0 (0.0) | 0.43 |
| ICD +/- CRT at randomization (%) | 71 (20.1) | 32 (23.5) | 26 (20.0) | 0.49 |
| Baseline BCIS jeopardy score, median (IQR)^b^ | 8 (6 to 10) | 10 (6 to 12) | 8 (6 to 10) | 0.0004 |
| Post-PCI BCIS jeopardy score, median (IQR) | - | 4 (2 to 6) | 0 (0 to 2) | <0.0001 |
| Baseline SYNTAX score, median (IQR) | 22 (15 to 29) | 26.0 (20.0 to 32.5) | 18.0 (12.0 to 23.0) | <0.0001 |
| Residual SYNTAX score, median (IQR) | - | 26.0 (20.0 to 32.5) | 18.0 (12.0 to 23.0) | <0.0001 |
| Total number of lesions, median (IQR) | 45 (12.8) | 3 (3 to 4) | 2 (2 to 3) | <0.0001 |
| Left main coronary artery disease (%) | 31.9 ± 9.6 | 28 (20.6) | 12 (9.2) | 0.009 |
| Left ventricular ejection fraction, mean (SD), %^c^ | 243 (77.1)  72 (22.9) | 31.1 ± 9.5 | 33.5 ± 10.4 | 0.07 |
| Viability test (%)  CMR  DSE | 7 (4-10) | 109 (80.1)  27 (19.9) | 101 (77.7)  29 (22.3) | - |
| Number of viable segments (IQR) |  | 6 (4-10) | 7 (5-11) | 0.29 |

^a^ Race as self-reported by participants using options defined by the investigators.

^b^ British Cardiovascular Intervention Society jeopardy score (BCIS-JS) as reported by angiography core laboratory.

^c^ Baseline left ventricular ejection fraction measured by the blinded echocardiography core laboratory

^d^ P-value denotes comparison between Incomplete vs complete anatomical revascularization groups

BCIS denotes British Cardiovascular Intervention Society, CABG coronary artery bypass grafting, CCS Canadian Cardiovascular Society, CMR cardiovascular magnetic resonance imaging, CRT cardiac resynchronization therapy, CTO chronic total occlusion, DSE dobutamine stress echocardiography, ICD implantable cardioverter defibrillator, IQR interquartile range, NYHA New York Heart Association, PCI percutaneous coronary intervention, RI_myo_ Myocardial revascularization index.

## Table S11 – Primary and secondary outcomes by viability guided completeness of revascularization (25% late gadolinium enhancement threshold)

|  | **Optimal medical therapy^§^ (Reference)** | **Complete viability guided revascularization^§^ (RI_myo_ >86.7)** | **Hazard / Odds ratio***  **(95% CI)** | **p-value** | **Incomplete viability guided revascularization^§^ (RI_myo_ ≤86.7)** | **Hazard / Odds ratio***  **(95% CI)** | **p-value** |
| --- | --- | --- | --- | --- | --- | --- | --- |
| All-cause death or hospitalization for heart failure | 134 (38.0) | 46 (34.8) | 1.02  (0.72 to 1.44) | 0.93 | 45 (33.8) | 0.79  (0.56 to 1.11) | 0.17 |
| All-cause death | 115 (32.6) | 40 (30.3) | 1.03  (0.71 to 1.50) | 0.88 | 36 (27.0) | 0.71  (0.48 to 1.04) | 0.08 |
| Cardiovascular death | 88 (24.9) | 27 (20.5) | 0.88  (0.56 to 1.37) | 0.56 | 26 (19.5) | 0.66  (0.42 to 1.03) | 0.07 |
| Hospitalization for heart failure | 54 (15.3) | 15 (11.4) | 0.80  (0.45 to 1.44) | 0.46 | 18 (13.5) | 0.76  (0.44 to 1.31) | 0.33 |
| Improvement in left ventricular ejection fraction | 101 (50.2) | 38 (48.1) | 0.94  (0.54 to 1.65) | 0.83 | 46 (55.4) | 1.02  (0.58 to 1.77) | 0.96 |

CI – confidence interval; HR-hazard ratio; RI_myo_ – Myocardial revascularization index

* Adjusted Hazard ratios calculated with OMT group as reference

**^§^** Event rate - n(%)

## Table S12 – Change in 2-year summary KCCQ score by anatomical and viability-guided completeness of revascularization

|  | **KCCQ* at baseline** | **KCCQ* at 2 years** | **Adjusted** difference in means at 2 years**  **(95% CI)** | **P-value** |
| --- | --- | --- | --- | --- |
| **Anatomical completeness of revascularization (RI_coro_)** |  |  |  |  |
| OMT | 63.0 (24.9) | 70.5 (24.7) | Reference |  |
| Incomplete anatomical revascularization (RI_coro_ ≤66.7%) | 57.8 (26.0) | 65.8 (26.2) | -1.1 (-6.1 to 3.9) | 0.66 |
| Complete anatomical revascularization (RI_coro_ >66.7%) | 65.8 (22.6) | 78.1 (22.9) | 4.6 (-0.2 to 9.5) | 0.06 |
|  |  |  |  |  |
| **Viability guided completeness of revasculariztion (RI_myo_)** |  |  |  |  |
| OMT | 63.0 (24.9) | 70.5 (24.7) | Reference |  |
| Incomplete viability guided revascularization (RI_myo_ ≤86.7%) | 60.6 (26.7) | 69.8 (26.3) | 0.1 (-4.8 to 5.0) | 0.97 |
| Complete viability guided revascularization (RI_myo_ >86.7%) | 63.2 (22.1) | 75.0 (24.0) | 3.9 (-0.9 to 8.6) | 0.11 |

CI – confidence interval; KCCQ – Kansas City Cardiomyopathy Questionnaire; OMT – optimal medical therapy; RI_coro_ – Coronary revascularization index; RI_myo_ – Myocardial revascularization index

Data are reported as mean (standard deviation); * KCCQ overall summary score; ** Adjusted for pre-specified adjustment variables and baseline KCCQ overall summary score.
